# Supplementary material for: The Impact of Amino Acids on Postprandial Glucose and Insulin Kinetics in Humans: A Quantitative Overview
Source: Nutrients. 2020 Oct 21;12(10):3211. doi: 10.3390/nu12103211 (PMC7594055; doi:10.3390/nu12103211)
Supplement: Supplementary file 1 [file nutrients-12-03211-s001.zip › Supplemental File 1.docx]

**SUPPLEMENTAL FILE 1:** PUBMED SEARCH STRATEGY

((“Amino Acids” [MeSH Terms] OR "Amino Acid" OR "Amino Acids" OR “Branched-Chain Amino Acids” OR “Branched-Chain Amino Acid” OR Alanine OR Arginine OR Asparagine OR “Aspartic Acid” OR Cysteine OR Glutamine OR “Glutamic Acid” OR Glycine OR Histidine OR Isoleucine OR Leucine OR Lysine OR Methionine OR Phenylalanine OR Proline OR Serine OR Threonine OR Tryptophan OR Tyrosine OR Valine OR Whey OR Casein) AND (Ingestion OR Ingested OR Infused OR Infusion OR Infusions OR Response OR Stimulation OR Elevation OR Elevated OR Oral OR Altered OR Alter OR Absorption OR Absorbed OR Postprandial OR Post-Prandial OR “Post Prandial” OR Supplemented OR Supplementation OR Supplement OR Eating OR Administration OR Administered OR Intravenous OR Intravenously OR Intake OR Food OR Consumption OR Consumed OR Dynamics OR Dynamic OR “Glucose Tolerance Test” OR “Tolerance Test” OR “Challenge Test”) AND (Insulinotropic OR Insulin OR Glucose OR Glycaemic OR Glycemia OR Glycaemia OR “Glycemic Control”) AND (Glucose [ti] OR Insulin [ti] OR Glycaemic [ti] OR Glycemia [ti] OR Glycaemia [ti] OR “Glycemic Control” [ti] OR “Amino Acids” [ti] OR “Amino Acid” [ti] OR Whey [ti] OR Casein [ti] OR Protein [ti] OR Proteins [ti] OR Alanine [ti] OR Arginine [ti] OR Asparagine [ti] OR “Aspartic Acid” [ti] OR Cysteine [ti] OR Glutamine [ti] OR “Glutamic Acid” [ti] OR Glycine [ti] OR Histidine [ti] OR Isoleucine [ti] OR Leucine [ti] OR Lysine [ti] OR Methionine [ti] OR Phenylalanine [ti] OR Proline [ti] OR Serine [ti] OR Threonine [ti] OR Tryptophan [ti] OR Tyrosine [ti] OR Valine [ti]) NOT Review [ptyp] AND English AND "loattrfull text"[sb] AND humans)
