# Supplementary material for: The Impact of Amino Acids on Postprandial Glucose and Insulin Kinetics in Humans: A Quantitative Overview
Source: Nutrients. 2020 Oct 21;12(10):3211. doi: 10.3390/nu12103211 (PMC7594055; doi:10.3390/nu12103211)
Supplement: Supplementary file 1 [file Supplemental Figure 3.pdf › Supplemental Figure 3.pdf]

Effect of glutamine intake on glucose and insulin dynamics

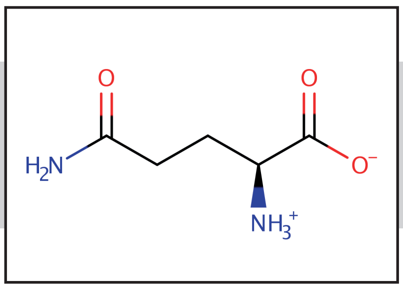

A Oral glutamine ingestion

First author	Subject condition	Amount administred	Ingestion type	# Subjects	Insulin time points	Insulin time to peak (min)	Insulin peak concentration (µU/mL)	Glucose time points	Glucose time to peak (min)	Glucose peak concentration (mg/dL)
[25] Greenfield, JR	Healthy	Water	Oral	8	5	0	4.3	7	0	83.2
	Healthy	30g glutamine	Oral	8	5	60	6.7	7	0	82.1
	T2DM	Water	Oral	8	5	15	25.1	7	0	112.1
	T2DM	30g glutamine	Oral	8	5	30	40.9	7	60	113.1
	Obese	Water	Oral	8	5	15	16.5	7	0	91.5
	Obese	30g glutamine	Oral	8	5	60	25.1	7	45	90.6

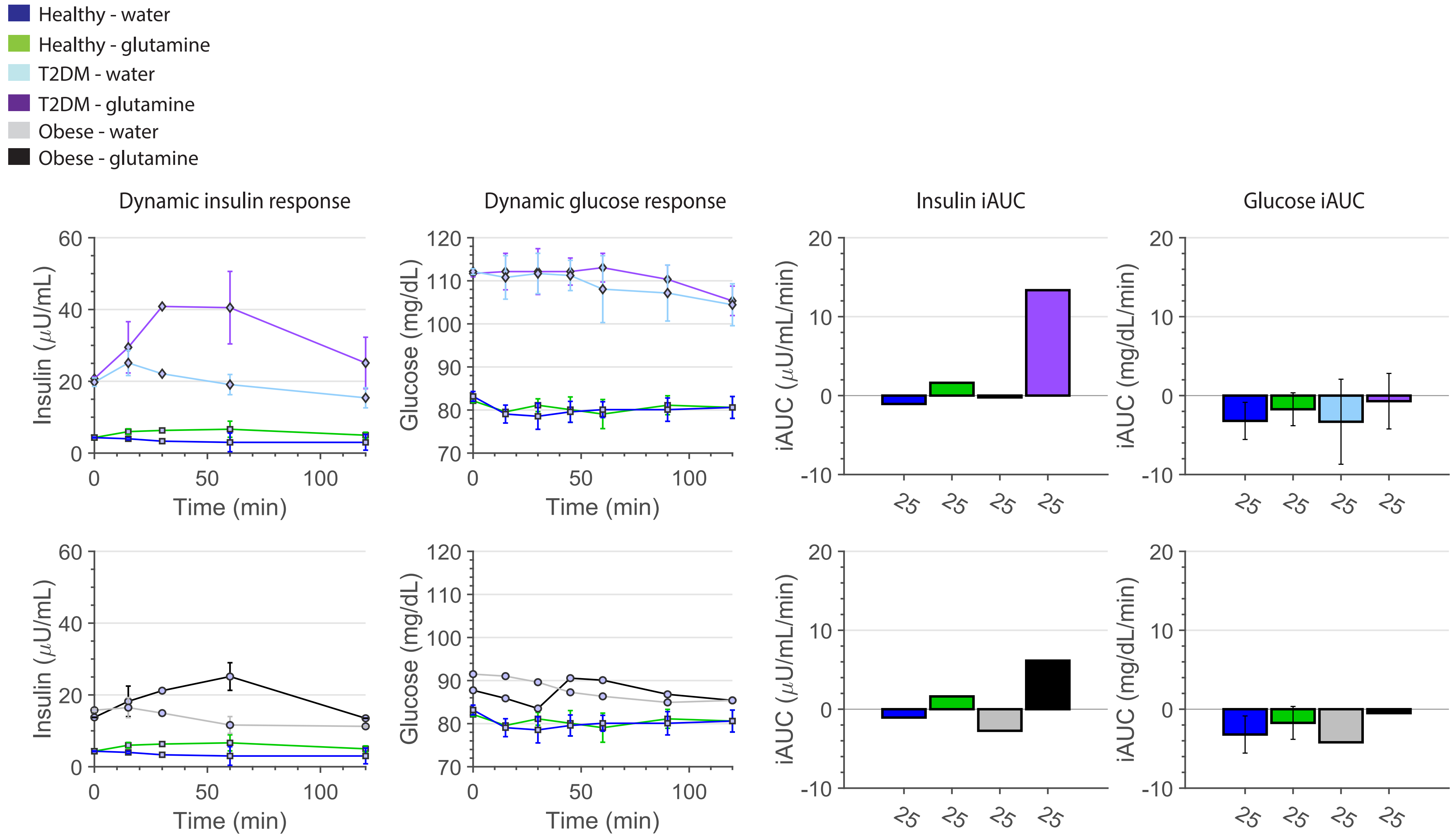

B Oral glutamine+glucose ingestion

First author	Subject condition	Amount administred	Ingestion type	# Subjects	Insulin time points	Insulin time to peak (min)	Insulin peak concentration (µU/mL)	Glucose time points	Glucose time to peak (min)	Glucose peak concentration (mg/dL)
No Data	No Data	No Data	No Data	No Data	No Data	No Data	No Data	No Data	No Data	No Data

C Intravenous glutamine infusion

First author	Subject condition	Amount administred	Ingestion type	# Subjects	Insulin time points	Insulin time to peak (min)	Insulin peak concentration (µU/mL)	Glucose time points	Glucose time to peak (min)	Glucose peak concentration (mg/dL)
No Data	No Data	No Data	No Data	No Data	No Data	No Data	No Data	No Data	No Data	No Data

*iAUC: Incremental area under the curve
**iAUC, time to peak and peak concentrations were calculated from the extracted data
