# Supplementary material for: The Impact of Amino Acids on Postprandial Glucose and Insulin Kinetics in Humans: A Quantitative Overview
Source: Nutrients. 2020 Oct 21;12(10):3211. doi: 10.3390/nu12103211 (PMC7594055; doi:10.3390/nu12103211)
Supplement: Supplementary file 1 [file nutrients-12-03211-s001.zip › Supplemental Figure 2.pdf]

Effect of alanine intake on glucose and insulin dynamics

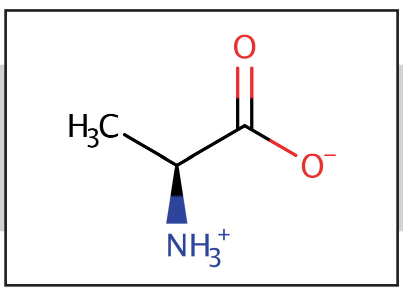

A Oral alanine ingestion

First author	Subject condition	Amount administred	Ingestion type	# Subjects	Insulin time points	Insulin time to peak (min)	Insulin peak concentration (μU/mL)	Glucose time points	Glucose time to peak (min)	Glucose peak concentration (mg/dL)
[19] Rossini, AA	Healthy	10g alanine	Oral	6	6	30	18.9	6	120	75.0
[20] Rose, DP	Healthy	11.9g alanine	Oral	13	7	30	18.2	7	0	88.0
[21] Sato, T[1]	Healthy	64.6g alanine	Oral	5	5	60	-	5	0	68.7
[22.1] Genuth, SM	Healthy	33.8g alanine	Oral	10	10	45	32.7	10	15	88.3
	T2DM	37.5g alanine	Oral	10	10	90	28.3	10	0	271.7
[22.2] Genuth, SM	Healthy	6.8g alanine	Oral	10	10	30	26.9	10	15	91.2
	T2DM	8.2g alanine	Oral	10	10	30	21.8	10	60	297.8
[23] Genuth, SM	Obese	50g alanine	Oral	6	9	45	214.8	9	0	114.4

Healthy - alanine
T2DM - alanine
Obese - alanine

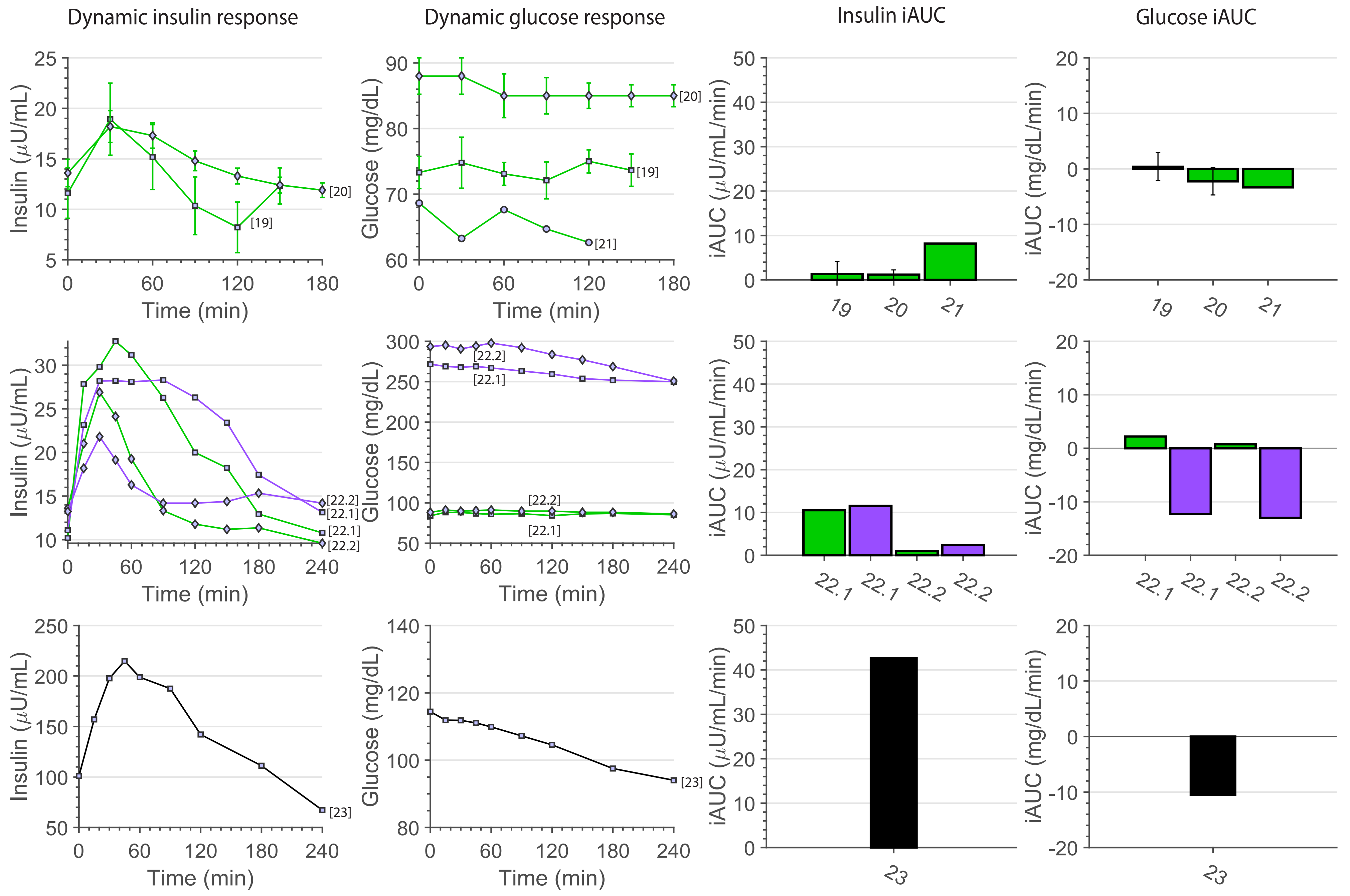

[1]Insulin time series data is not shown due to only incremental values provided

B Oral alanine+glucose ingestion

First author	Subject condition	Amount administred	Ingestion type	# Subjects	Insulin time points	Insulin time to peak (min)	Insulin peak concentration (μU/mL)	Glucose time points	Glucose time to peak (min)	Glucose peak concentration (mg/dL)
No Data	No Data	No Data	No Data	No Data	No Data	No Data	No Data	No Data	No Data	No Data

C Intravenous alanine infusion

First author	Subject condition	Amount administred	Ingestion type	# Subjects	Insulin time points	Insulin time to peak (min)	Insulin peak concentration (μU/mL)	Glucose time points	Glucose time to peak (min)	Glucose peak concentration (mg/dL)
[19] Rossini, AA	Healthy	10g alanine	IV-infusion	6	6	30	13.9	6	30	75.7
[24] Asano, T	Healthy	5.3g alanine	IV-infusion	9	10	5	17.9	10	5	92.0
	Obese	8.9g alanine	IV-infusion	6	10	20	36.3	10	20	90.7

Healthy - alanine
Obese - alanine

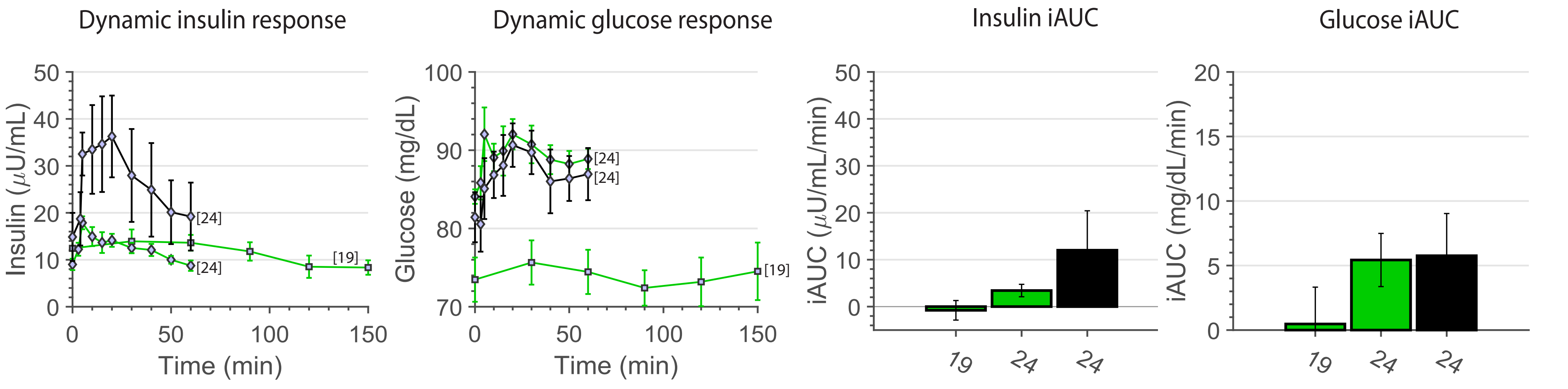

*iAUC: Incremental area under the curve
**iAUC, time to peak and peak concentrations were calculated from the extracted data
