# Supplementary material for: The Impact of Amino Acids on Postprandial Glucose and Insulin Kinetics in Humans: A Quantitative Overview
Source: Nutrients. 2020 Oct 21;12(10):3211. doi: 10.3390/nu12103211 (PMC7594055; doi:10.3390/nu12103211)
Supplement: Supplementary file 1 [file nutrients-12-03211-s001.zip › Supplemental Figure 4.pdf]

Effect of arginine intake on glucose and insulin dynamics

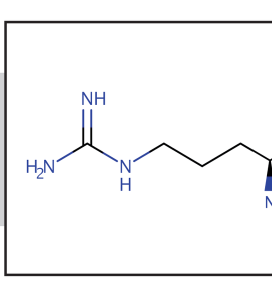

A — Oral arginine ingestion

First author	Subject condition	Amount administred	Ingestion type	# Subjects	Insulin time points	Insulin time to peak (min)	Insulin peak concentration (µU/mL)	Glucose time points	Glucose time to peak (min)	Glucose peak concentration (mg/dL)
[26] Gannon, MC	Healthy	Water	Oral	9	13	120	7.9	13	70	78.7
	Healthy	10.6g arginine	Oral	9	13	10	13.6	13	120	81.3
[27] Tang, ZQ	Healthy	Water	Oral	8	6	15	2.5	6	30	96.9
	Healthy	30g arginine	Oral	8	6	45	5.4	6	15	82.8

Healthy - water
Healthy - arginine

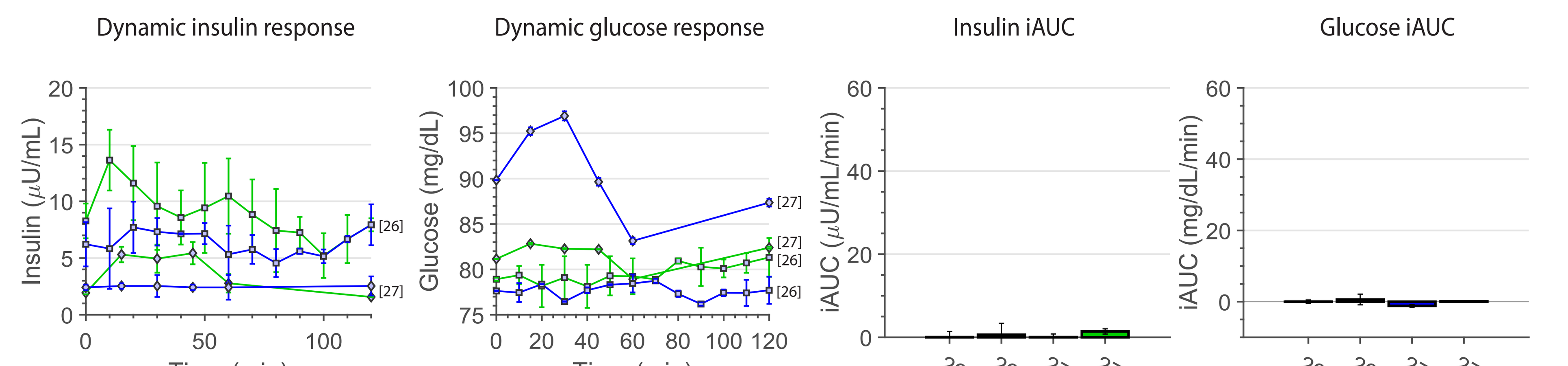

B — Oral arginine+glucose ingestion

First author	Subject condition	Amount administred	Ingestion type	# Subjects	Insulin time points	Insulin time to peak (min)	Insulin peak concentration (µU/mL)	Glucose time points	Glucose time to peak (min)	Glucose peak concentration (mg/dL)
[26] Gannon, MC	Healthy	25g glucose	Oral	9	13	30	93.8	13	30	124.2
	Healthy	10.6g arginine +25g glucose	Oral	9	13	30	65.8	13	30	96.1
[27] Tang, ZQ	Healthy	75g glucose	Oral	8	6	45	39.8	6	45	135.1
	Healthy	30g arginine +75g glucose	Oral	8	6	30	42.2	6	30	130.9

Healthy - glucose
Healthy - arginine+glucose

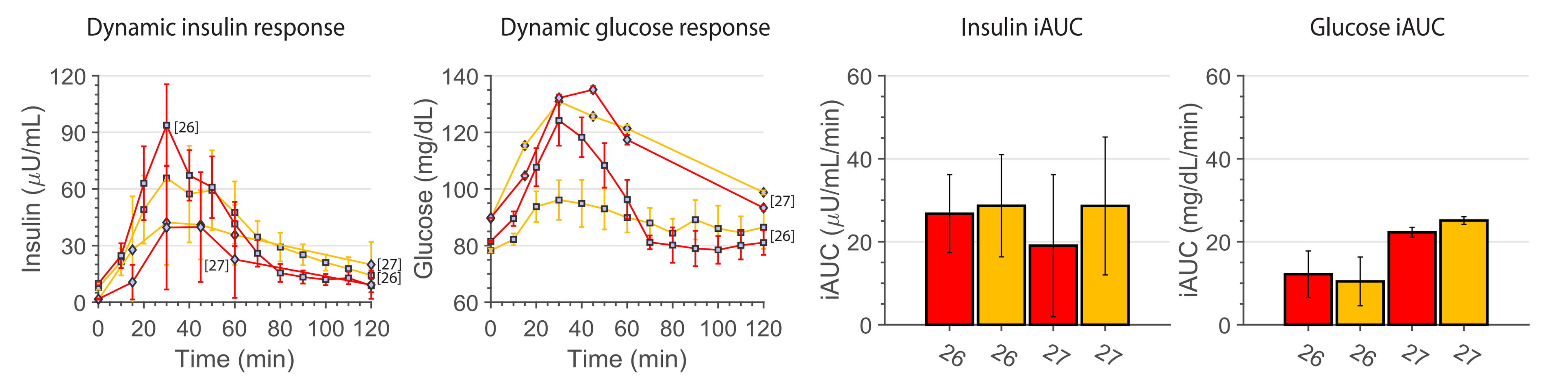

C — Intravenous arginine infusion

First author	Subject condition	Amount administred	Ingestion type	# Subjects	Insulin time points	Insulin time to peak (min)	Insulin peak concentration (µU/mL)	Glucose time points	Glucose time to peak (min)	Glucose peak concentration (mg/dL)
[17] Floyd, JC	Healthy	15g arginine	IV-Infusion	11	10	5	35.1	10	30	104.2
[28] Giugliano, D	Healthy	30g arginine	IV-Infusion	10	9	30	34.2	9	25	107.8
[29] Coiro, V	Healthy	30g arginine	IV-Infusion	14	9	30	52.8	9	20	108.8
[30] Broglio, F [1]	Healthy	Saline	IV-Infusion	7	9	15	-	9	0	-
	Healthy	34g arginine	IV-Infusion	7	9	30	-	9	30	-
[31.1] Dela, F	Healthy-untrained	68.4g arginine	IV-Infusion	7	11	3	83.8	16	20	129.2
[31.2] Dela, F	Healthy-trained	63.0g arginine	IV-Infusion	7	11	30	35.2	16	20	124.2
[32] Penny, R	Healthy	Saline	IV-Infusion	8	7	90	30.9	7	120	88.8
	Healthy	34g arginine	IV-Infusion	8	7	30	101.0	7	30	91.7
[33.1] Levin, SR	Healthy	20g arginine	IV-Infusion	6	7	20	34.7	7	20	93.5
[33.2] Levin, SR	Healthy	20g arginine	IV-Infusion	6	7	30	31.2	7	20	89.8
[34] Bratusch-Marrain, P	Healthy	30g arginine	IV-Infusion	9	9	30	97.5	13	25	95.5
[35] Imura, H	Healthy	30g arginine	IV-Infusion	15	9	45	45.6	9	30	108.6
[36] Hasselblatt, M	Healthy	30g arginine	IV-Infusion	15	8	30	31.4	8	30	105.1
[37] Berger, V	Healthy	30g arginine	IV-Infusion	9	6	30	40.1	5	20	90.0
[38.1] Dupre, J [1]	Healthy	15g arginine	IV-Infusion	13	6	30	30.2	6	20	19.0
[38.2] Dupre, J [1]	Healthy	3.9g arginine	IV-Infusion	6	6	5	29.0	6	40	4.8
[39.1] Dupre, J	Healthy	15g arginine	IV-Infusion	4	7	30	52.5	7	30	90.0
[39.2] Dupre, J	Healthy	15g arginine	IV-Infusion	6	7	20	44.0	7	20	93.6
[40] Kimber, JL	Healthy	31.8g arginine	IV-Infusion	5	7	15	11.2	7	30	86.5
[41] Ohneda	Healthy	30g arginine	IV-Infusion	8	5	15	44.8	5	30	75.9

Healthy - saline
Healthy - arginine

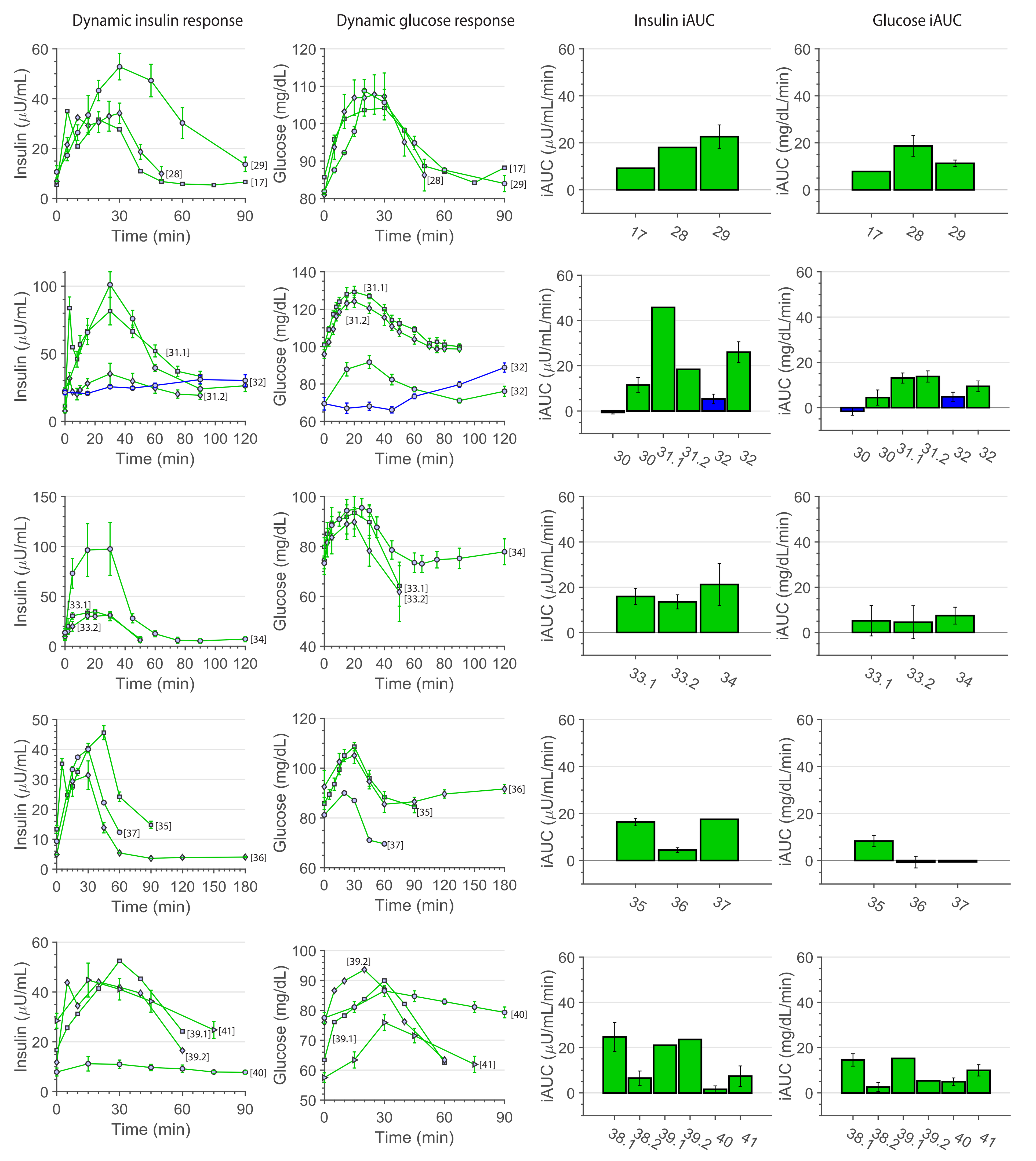

[1] Insulin and glucose time series data are not shown due to only incremental values provided

First author	Subject condition	Amount administred	Ingestion type	# Subjects	Insulin time points	Insulin time to peak (min)	Insulin peak concentration (µU/mL)	Glucose time points	Glucose time to peak (min)	Glucose peak concentration (mg/dL)
[42] Efendic, SE	Healthy	27.9g arginine	IV-Infusion	7	7	5	56.1	7	10	80.2
	T2DM	29.8g arginine	IV-Infusion	6	7	30	51.7	7	30	119.4
[43] Kawamori, R	Healthy	32.3g arginine	IV-Infusion	9	9	30	81.8	9	30	125.7
	T2DM	33.5g arginine	IV-Infusion	5	10	2	20.5	9	45	272.9
[44] Sparks, LL	Healthy	30g arginine	IV-Infusion	10	5	30	145.0	5	30	92.0
	T2DM	30g arginine	IV-Infusion	6	4	30	49.0	5	30	109.0
[45] Kawamori, R	Healthy	32.3g arginine	IV-Infusion	9	9	30	79.5	9	30	128.6
	T2DM	32.3g arginine	IV-Infusion	7	9	45	21.5	9	45	270.3
[46] Maccario, M	Healthy	27.2g arginine	IV-Infusion	7	10	15	34.5	9	15	86.5
	Obese	50.6g arginine	IV-Infusion	7	10	30	66.0	9	15	100.3
[47] Marfella, R	T2DM	30g arginine	IV-Infusion	10	9	30	45.4	9	10	155.0
[48] Raskin, P	T2DM	20g arginine	IV-Infusion	6	5	40	26.3	5	40	234.0
[49] Maejima, K	T2DM	30g arginine	IV-Infusion	12	7	30	24.4	7	45	155.0
[50] Ohneda, P	T2DM	30g arginine	IV-Infusion	5	10	40	31.9	10	30	174.1
[51] Carpentier, AA	Obese	30g arginine	IV-Infusion	14	14	3	81.8	14	30	116.9
[52] Maccario, M	Obese	49.3g arginine	IV-Infusion	6	12	30	69.4	12	30	100.6
[53] Walter, RM	Obese	5g arginine	IV-Infusion	5	9	2	97.0	9	8	154.0

Healthy - arginine
T2DM - arginine
Obese - arginine

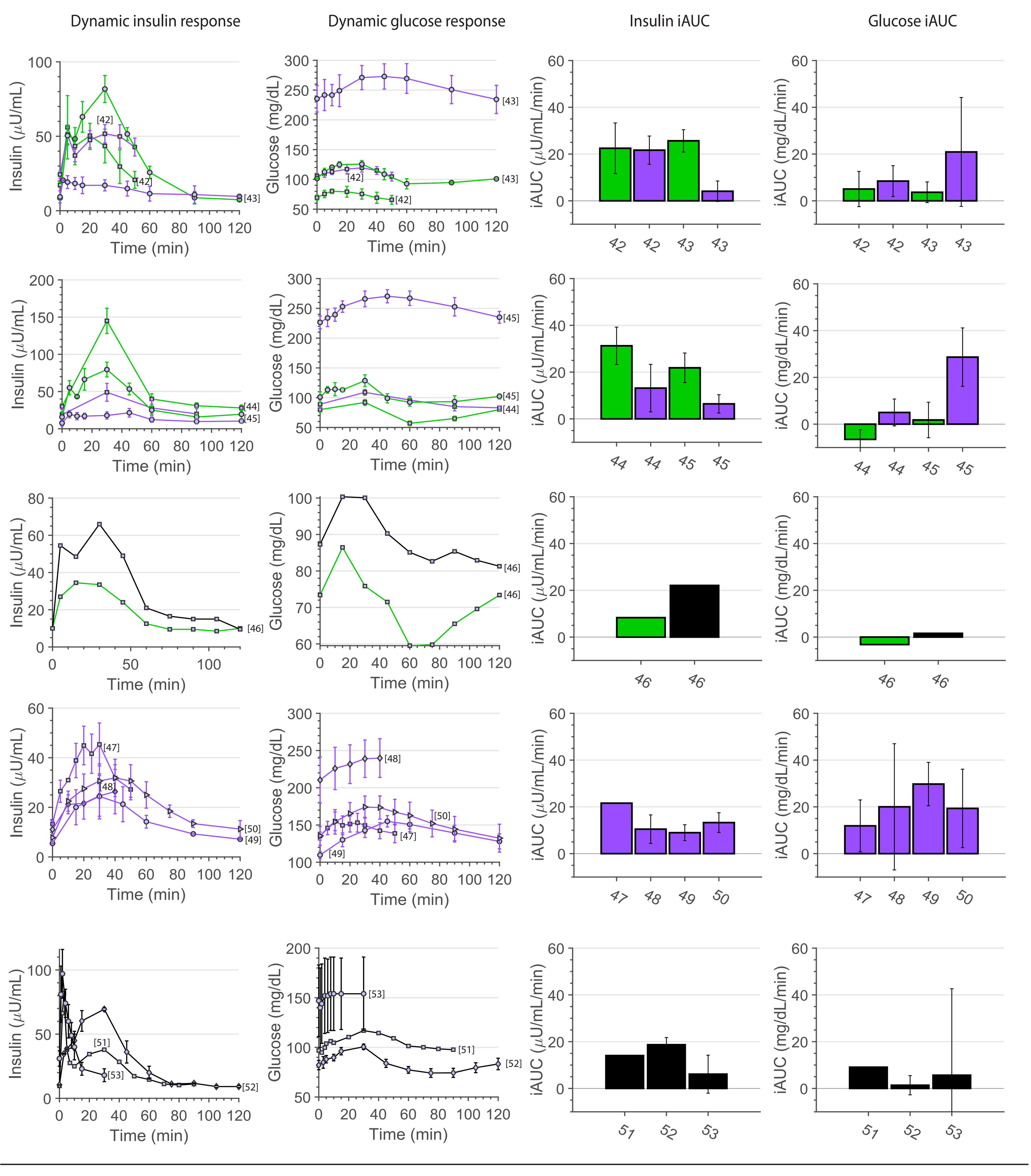

*iAUC: Incremental area under the curve
**iAUC, time to peak and peak concentrations were calculated from the extracted data
