# Supplementary material for: The Impact of Amino Acids on Postprandial Glucose and Insulin Kinetics in Humans: A Quantitative Overview
Source: Nutrients. 2020 Oct 21;12(10):3211. doi: 10.3390/nu12103211 (PMC7594055; doi:10.3390/nu12103211)
Supplement: Supplementary file 1 [file nutrients-12-03211-s001.zip › Supplemental Figure 5.pdf]

Effect of lysine intake on glucose and insulin dynamics

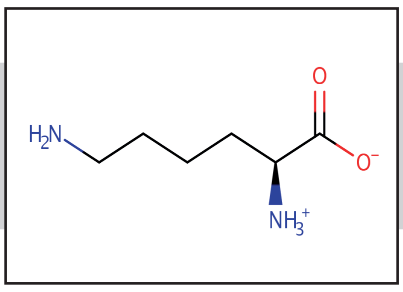

A Oral lysine ingestion

First author	Subject condition	Amount administred	Ingestion type	# Subjects	Insulin time points	Insulin time to peak (min)	Insulin peak concentration (µU/mL)	Glucose time points	Glucose time to peak (min)	Glucose peak concentration (mg/dL)
[54] Kalogeropoulou, D	Healthy	Water	Oral	13	14	20	7.0	14	150	81.7
	Healthy	11g lysine	Oral	13	14	10	9.8	14	150	86.0

Healthy - water
Healthy - lysine

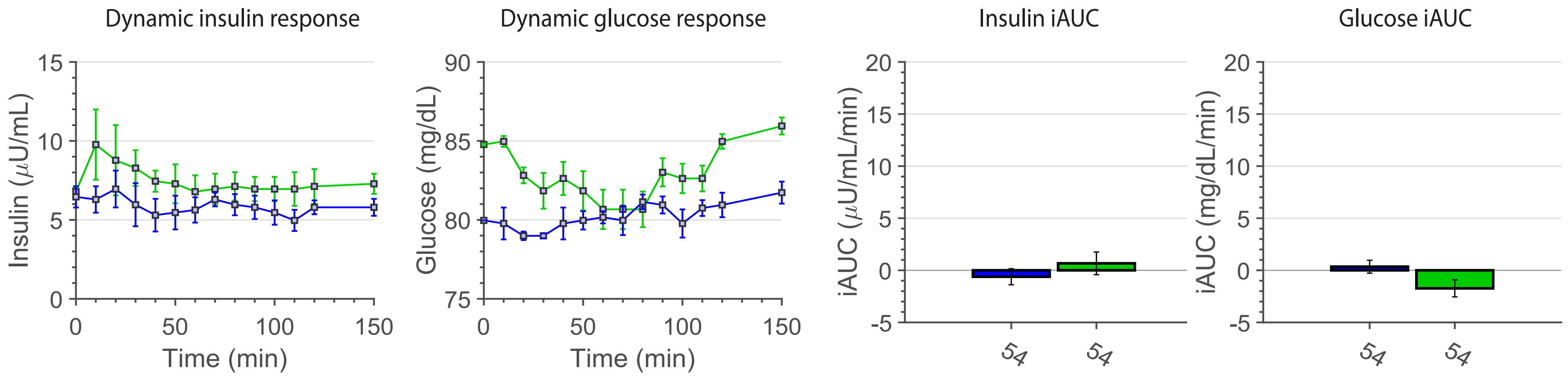

B Oral lysine+glucose ingestion

First author	Subject condition	Amount administred	Ingestion type	# Subjects	Insulin time points	Insulin time to peak (min)	Insulin peak concentration (µU/mL)	Glucose time points	Glucose time to peak (min)	Glucose peak concentration (mg/dL)
[54] Kalogeropoulou, D	Healthy	25g glucose	Oral	13	14	30	35.4	14	40	124.0
	Healthy	11g lysine +25g glucose	Oral	13	14	30	38.7	14	30	109.9

Healthy - glucose
Healthy - lysine+glucose

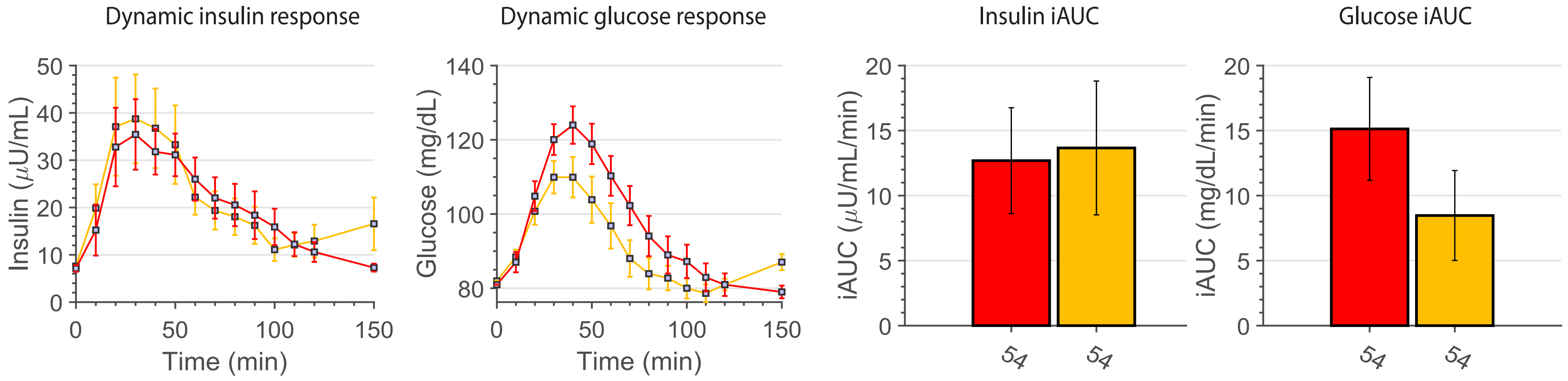

C Intravenous lysine infusion

First author	Subject condition	Amount administred	Ingestion type	# Subjects	Insulin time points	Insulin time to peak (min)	Insulin peak concentration (µU/mL)	Glucose time points	Glucose time to peak (min)	Glucose peak concentration (mg/dL)
[17] Floyd, JC	Healthy	15g lysine	IV-infusion	11	10	5	37.0	10	5	97.7

Healthy - lysine

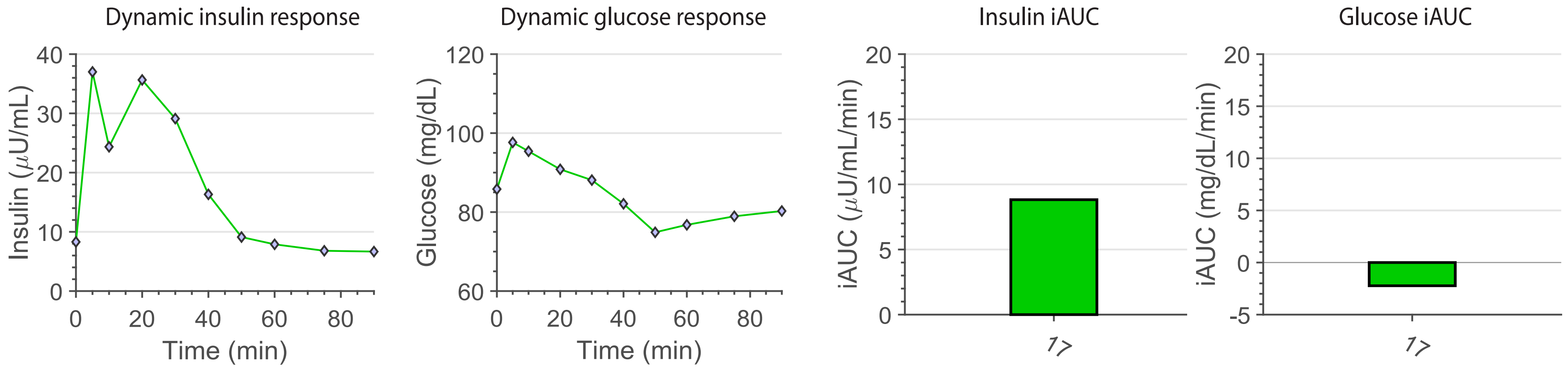

*iAUC: Incremental area under the curve
**iAUC, time to peak and peak concentrations were calculated from the extracted data
