# Supplementary material for: The Impact of Amino Acids on Postprandial Glucose and Insulin Kinetics in Humans: A Quantitative Overview
Source: Nutrients. 2020 Oct 21;12(10):3211. doi: 10.3390/nu12103211 (PMC7594055; doi:10.3390/nu12103211)
Supplement: Supplementary file 1 [file nutrients-12-03211-s001.zip › Supplemental Figure 6.pdf]

Effect of glycine intake on glucose and insulin dynamics

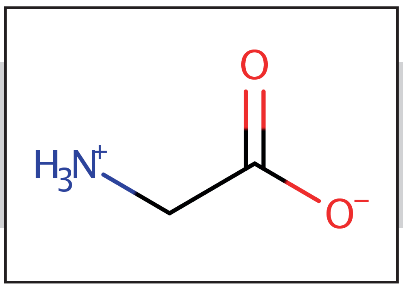

A Oral glycine ingestion

First author	Subject condition	Amount administred	Ingestion type	# Subjects	Insulin time points	Insulin time to peak (min)	Insulin peak concentration (µU/mL)	Glucose time points	Glucose time to peak (min)	Glucose peak concentration (mg/dL)
[55] Gannon, MC	Healthy	Water	Oral	9	13	0	7.5	13	120	78.9
	Healthy	4.6g glycine	Oral	9	13	30	14.0	13	120	80.0
[56] Kasai, K	Healthy	22.5g glycine	Oral	19	10	15	25.9	10	45	85.9

Healthy - water
Healthy - glycine

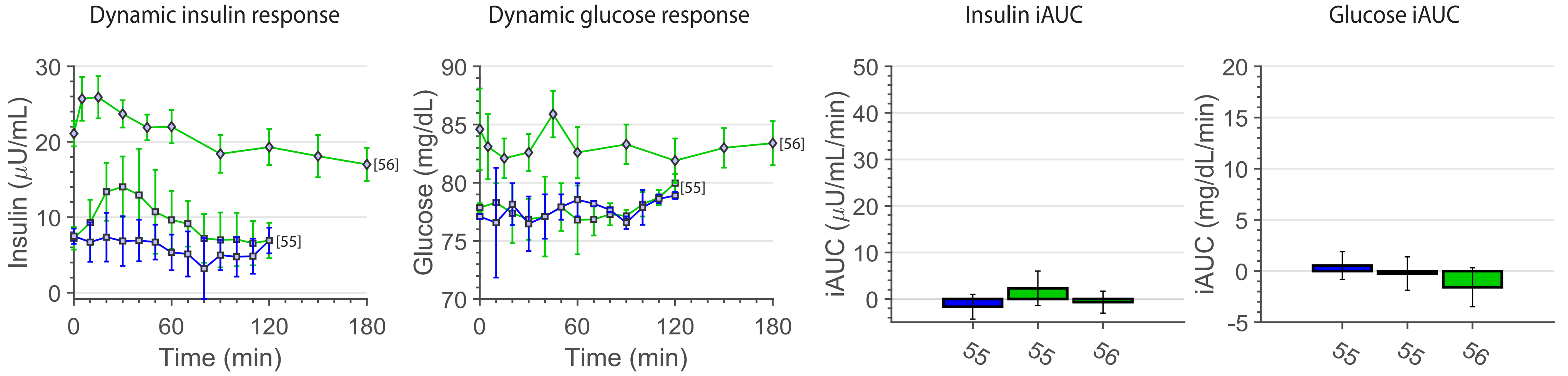

B Oral glycine+glucose ingestion

First author	Subject condition	Amount administred	Ingestion type	# Subjects	Insulin time points	Insulin time to peak (min)	Insulin peak concentration (µU/mL)	Glucose time points	Glucose time to peak (min)	Glucose peak concentration (mg/dL)
[55] Gannon, MC	Healthy	25g glucose	Oral	9	13	30	92.4	13	30	123.9
	Healthy	4.6g glycine +25g glucose	Oral	9	13	40	76.7	13	40	105.3

Healthy - glucose
Healthy - glycine+glucose

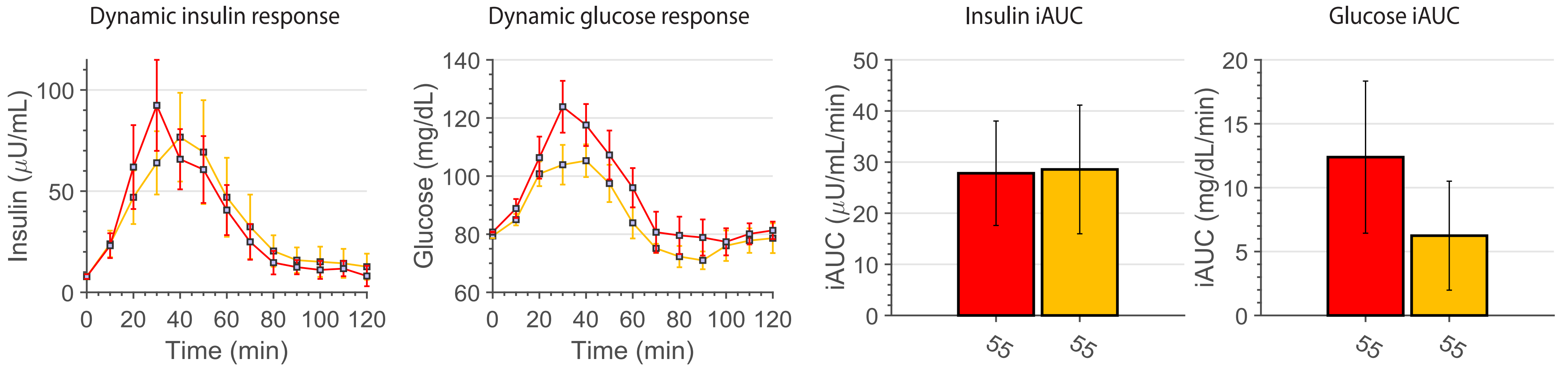

C Intravenous glycine infusion

First author	Subject condition	Amount administred	Ingestion type	# Subjects	Insulin time points	Insulin time to peak (min)	Insulin peak concentration (µU/mL)	Glucose time points	Glucose time to peak (min)	Glucose peak concentration (mg/dL)
No Data	No Data	No Data	No Data	No Data	No Data	No Data	No Data	No Data	No Data	No Data

*iAUC: Incremental area under the curve
**iAUC, time to peak and peak concentrations were calculated from the extracted data
