# Supplementary material for: The Impact of Amino Acids on Postprandial Glucose and Insulin Kinetics in Humans: A Quantitative Overview
Source: Nutrients. 2020 Oct 21;12(10):3211. doi: 10.3390/nu12103211 (PMC7594055; doi:10.3390/nu12103211)
Supplement: Supplementary file 1 [file nutrients-12-03211-s001.zip › Supplemental Figure 7.pdf]

Effect of proline intake on glucose and insulin dynamics

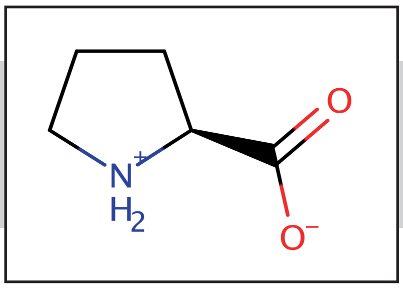

A — Oral proline ingestion

First author	Subject condition	Amount administred	Ingestion type	# Subjects	Insulin time points	Insulin time to peak (min)	Insulin peak concentration (µU/mL)	Glucose time points	Glucose time to peak (min)	Glucose peak concentration (mg/dL)
[57] Nuttal, FQ	Healthy	Water	Oral	8	14	30	7.9	14	110	81.4
	Healthy	6g proline	Oral	8	14	30	11.6	14	150	82.6

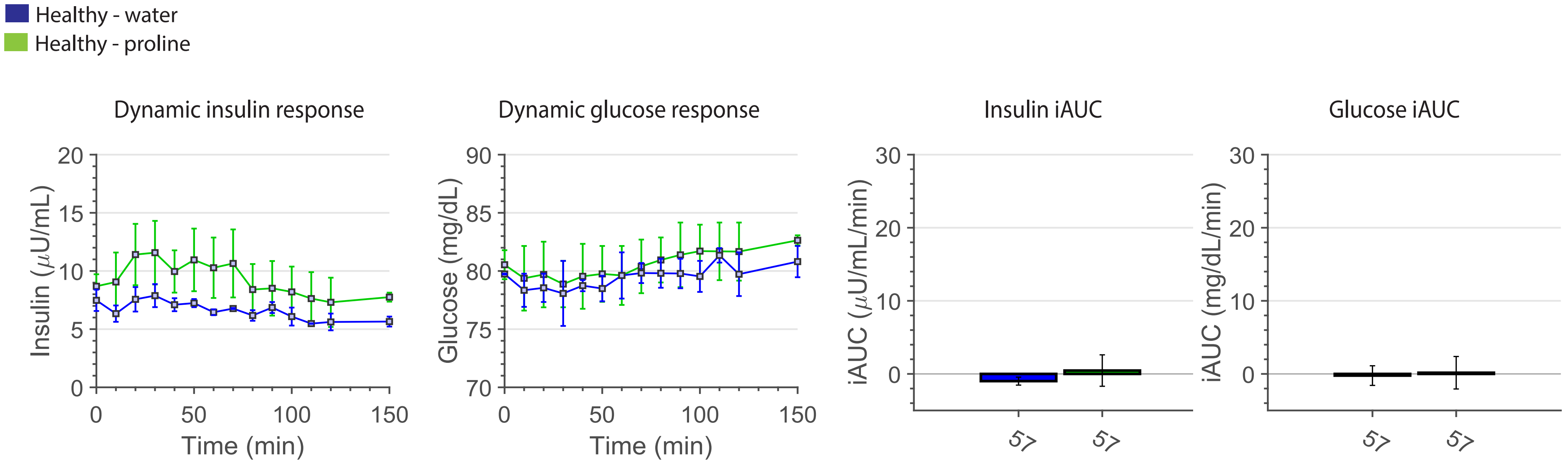

B — Oral proline+glucose ingestion

First author	Subject condition	Amount administred	Ingestion type	# Subjects	Insulin time points	Insulin time to peak (min)	Insulin peak concentration (µU/mL)	Glucose time points	Glucose time to peak (min)	Glucose peak concentration (mg/dL)
[57] Nuttal, FQ	Healthy	25g glucose	Oral	8	14	30	33.0	14	60	126.1
	Healthy	6g proline +25g glucose	Oral	8	14	40	41.2	14	30	110.6

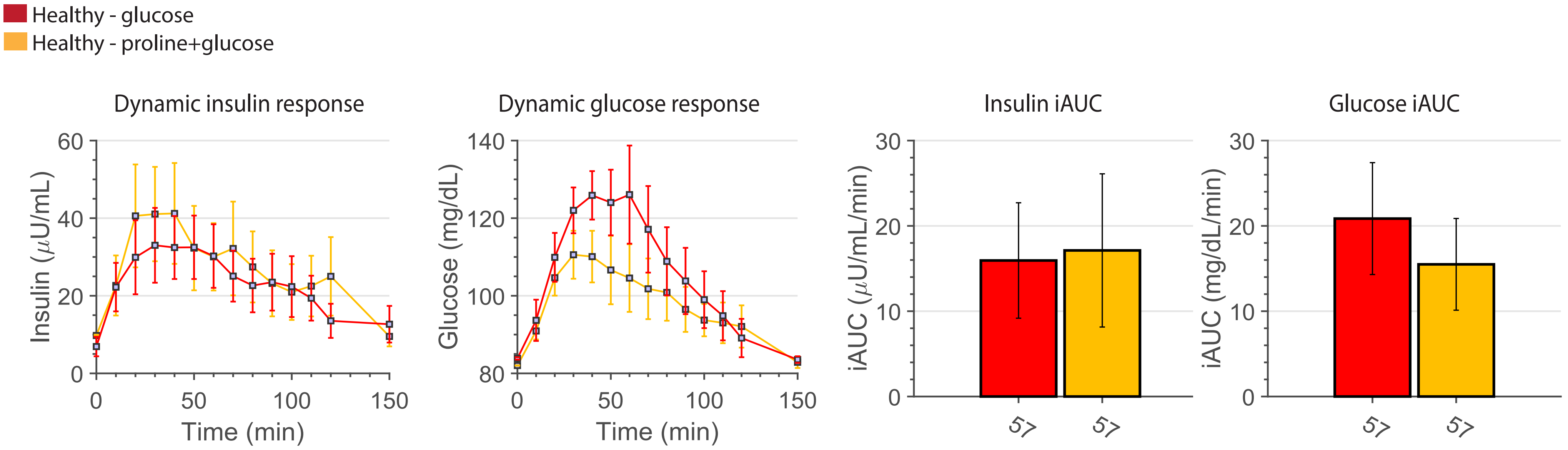

C — Intravenous proline infusion

First author	Subject condition	Amount administred	Ingestion type	# Subjects	Insulin time points	Insulin time to peak (min)	Insulin peak concentration (µU/mL)	Glucose time points	Glucose time to peak (min)	Glucose peak concentration (mg/dL)
No Data	No Data	No Data	No Data	No Data	No Data	No Data	No Data	No Data	No Data	No Data

*iAUC: Incremental area under the curve

**iAUC, time to peak and peak concentrations were calculated from the extracted data
