# Supplementary material for: The Impact of Amino Acids on Postprandial Glucose and Insulin Kinetics in Humans: A Quantitative Overview
Source: Nutrients. 2020 Oct 21;12(10):3211. doi: 10.3390/nu12103211 (PMC7594055; doi:10.3390/nu12103211)
Supplement: Supplementary file 1 [file nutrients-12-03211-s001.zip › Supplemental Figure 8.pdf]

Effect of phenylalanine intake on glucose and insulin dynamics

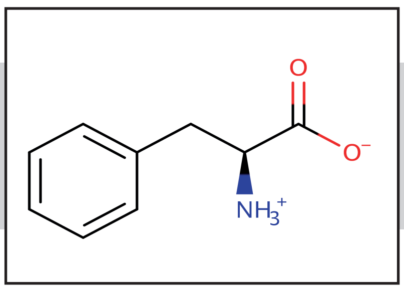

A Oral phenylalanine ingestion

First author	Subject condition	Amount administred	Ingestion type	# Subjects	Insulin time points	Insulin time to peak (min)	Insulin peak concentration (μU/mL)	Glucose time points	Glucose time to peak (min)	Glucose peak concentration (mg/dL)
[58] Nuttal, FQ	Healthy	Water	Oral	6	14	20	9.9	14	100	80.0
	Healthy	9.7g phenylalanine	Oral	6	14	30	14.4	14	60	84.2

Healthy - water
Healthy - phenylalanine

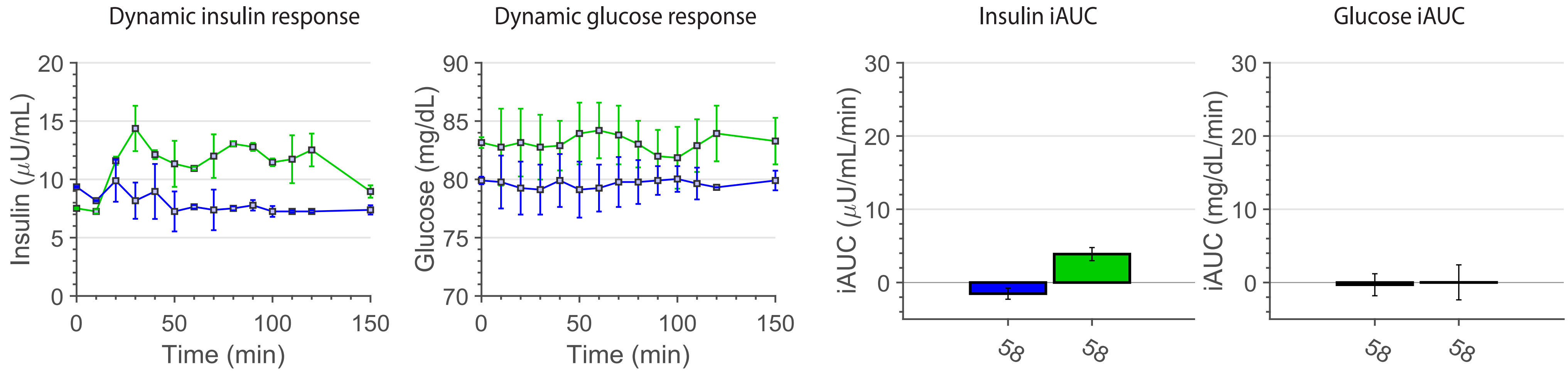

B Oral phenylalanine+glucose ingestion

First author	Subject condition	Amount administred	Ingestion type	# Subjects	Insulin time points	Insulin time to peak (min)	Insulin peak concentration (μU/mL)	Glucose time points	Glucose time to peak (min)	Glucose peak concentration (mg/dL)
[58] Nuttal, FQ	Healthy	25g glucose	Oral	6	14	50	49.3	14	40	109.8
	Healthy	9.7g phenylalanine +25g glucose	Oral	6	14	30	64.8	14	30	112.7

Healthy - glucose
Healthy - phenylalanine+glucose

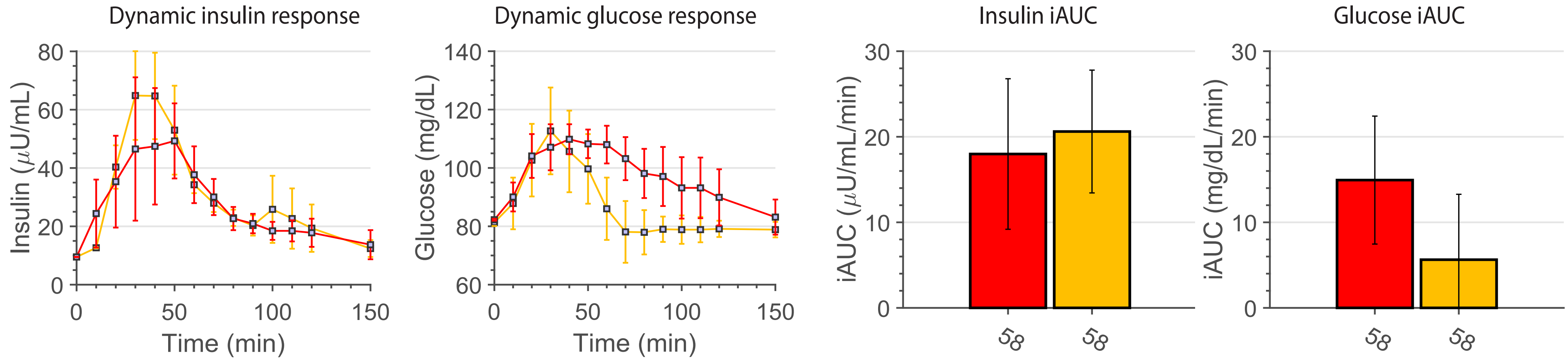

C Intravenous phenylalanine infusion

First author	Subject condition	Amount administred	Ingestion type	# Subjects	Insulin time points	Insulin time to peak (min)	Insulin peak concentration (μU/mL)	Glucose time points	Glucose time to peak (min)	Glucose peak concentration (mg/dL)
[17] Floyd, JC	Healthy	15g phenylalanine	IV-Infusion	11	10	5	20.2	10	0	93.4

Healthy - phenylalanine

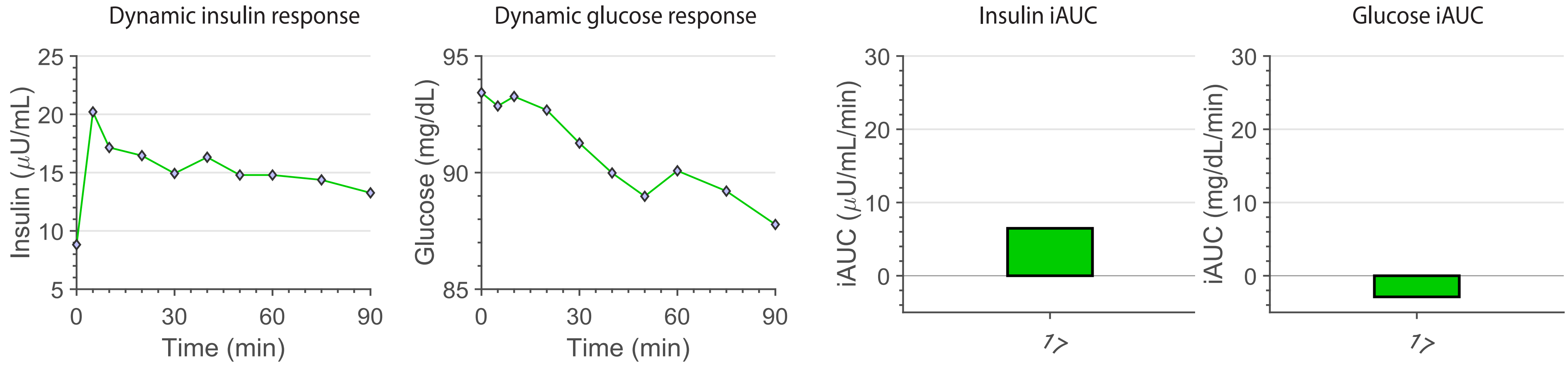

*iAUC: Incremental area under the curve
**iAUC, time to peak and peak concentrations were calculated from the extracted data
