# Supplementary material for: The Impact of Amino Acids on Postprandial Glucose and Insulin Kinetics in Humans: A Quantitative Overview
Source: Nutrients. 2020 Oct 21;12(10):3211. doi: 10.3390/nu12103211 (PMC7594055; doi:10.3390/nu12103211)
Supplement: Supplementary file 1 [file nutrients-12-03211-s001.zip › Supplemental Figure 9.pdf]

Effect of glutamate intake on glucose and insulin dynamics

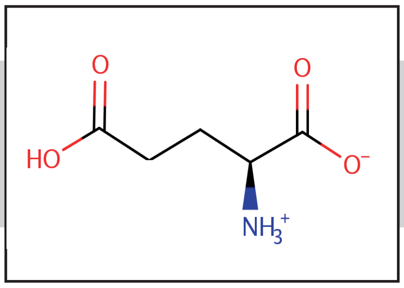

A Oral glutamate ingestion

First author	Subject condition	Amount administred	Ingestion type	# Subjects	Insulin time points	Insulin time to peak (min)	Insulin peak concentration (µU/mL)	Glucose time points	Glucose time to peak (min)	Glucose peak concentration (mg/dL)
[59] Di Sibastiano, KM	Healthy	Control	Oral	9	11	40	5.5	11	30	82.8
	Healthy	11.9 glutamate	Oral	9	11	40	9.0	11	10	81.1
[60] Fernstrom, JD[1]	Healthy	Control	Oral	8	13	0	5.2	-	-	-
	Healthy	12.7g glutamate	Oral	8	13	20	11.6	-	-	-
[61] Graham, TE[1]	Healthy	11.5g glutamate	Oral	9	8	15	11.2	-	-	-

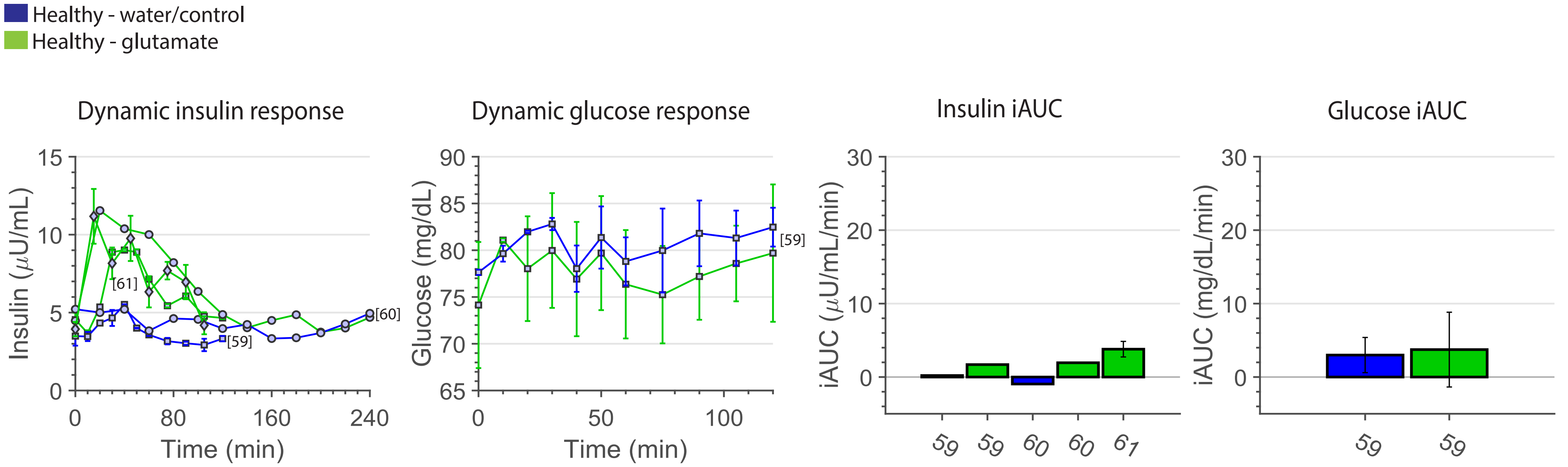

[1]Glucose values were not reported

B Oral glutamate+glucose ingestion

First author	Subject condition	Amount administred	Ingestion type	# Subjects	Insulin time points	Insulin time to peak (min)	Insulin peak concentration (µU/mL)	Glucose time points	Glucose time to peak (min)	Glucose peak concentration (mg/dL)
No Data	No Data	No Data	No Data	No Data	No Data	No Data	No Data	No Data	No Data	No Data

C Intravenous glutamate infusion

First author	Subject condition	Amount administred	Ingestion type	# Subjects	Insulin time points	Insulin time to peak (min)	Insulin peak concentration (µU/mL)	Glucose time points	Glucose time to peak (min)	Glucose peak concentration (mg/dL)
No Data	No Data	No Data	No Data	No Data	No Data	No Data	No Data	No Data	No Data	No Data

*iAUC: Incremental area under the curve
**iAUC, time to peak and peak concentrations were calculated from the extracted data
