# Supplementary material for: The Impact of Amino Acids on Postprandial Glucose and Insulin Kinetics in Humans: A Quantitative Overview
Source: Nutrients. 2020 Oct 21;12(10):3211. doi: 10.3390/nu12103211 (PMC7594055; doi:10.3390/nu12103211)
Supplement: Supplementary file 1 [file nutrients-12-03211-s001.zip › Supplemental Figure 10.pdf]

Effect of BCAA intake on glucose and insulin dynamics

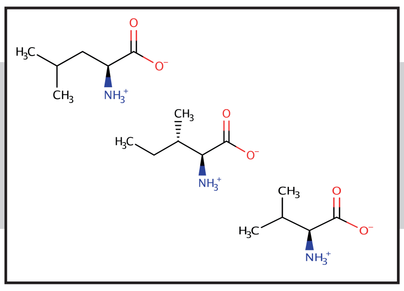

A Oral BCAA ingestion

First author	Subject condition	Amount administred	Ingestion type	# Subjects	Insulin time points	Insulin time to peak (min)	Insulin peak concentration (µU/mL)	Glucose time points	Glucose time to peak (min)	Glucose peak concentration (mg/dL)
[62.1] Zhang, Y	Healthy	1g BCAA	Oral	5	8	15	7.3	8	0	95.0
[62.2] Zhang, Y	Healthy	5g BCAA	Oral	5	8	15	8.5	8	15	96.0
[63] Gojda, J	Healthy	Control	Oral	18	11	45	6.6	11	180	83.3
	Healthy	30.7g BCAA	Oral	18	11	60	12.0	11	0	83.7

Healthy - control
Healthy - BCAA

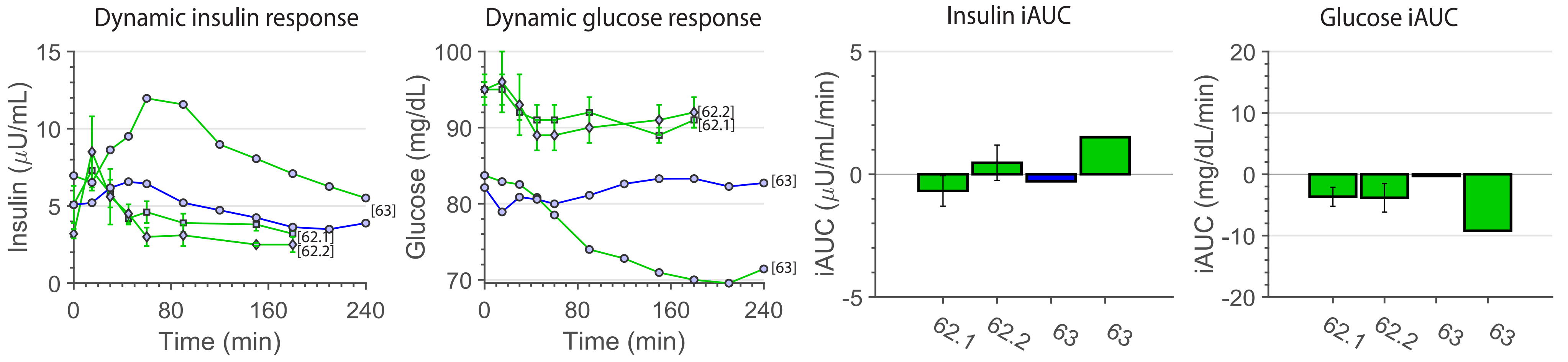

B Oral BCAA+glucose ingestion

First author	Subject condition	Amount administred	Ingestion type	# Subjects	Insulin time points	Insulin time to peak (min)	Insulin peak concentration (µU/mL)	Glucose time points	Glucose time to peak (min)	Glucose peak concentration (mg/dL)
No Data	No Data	No Data	No Data	No Data	No Data	No Data	No Data	No Data	No Data	No Data

C Intravenous BCAA infusion

First author	Subject condition	Amount administred	Ingestion type	# Subjects	Insulin time points	Insulin time to peak (min)	Insulin peak concentration (µU/mL)	Glucose time points	Glucose time to peak (min)	Glucose peak concentration (mg/dL)
[63] Gojda, J	Healthy	30.7g BCAA	IV-Infusion	18	11	15	10.1	11	0	84.0
[64.1] Tatpati, LL	Healthy (Young)	Saline	IV-Infusion	12	5	0	4.4	9	0	94.1
	Healthy (Young)	18.5g BCAA	IV-Infusion	12	5	120	6.2	9	0	92.2
[64.2] Tatpati, LL	Healthy (Elderly)	Saline	IV-Infusion	12	5	0	4.4	9	0	105.0
	Healthy (Elderly)	16.35g BCAA	IV-Infusion	12	5	120	4.7	9	0	102.8
[65] Louard, RJ	Healthy	3.4g BCAA	IV-Infusion	10	5	180	8.6	7	0	86.8

Healthy - saline
Healthy - BCAA

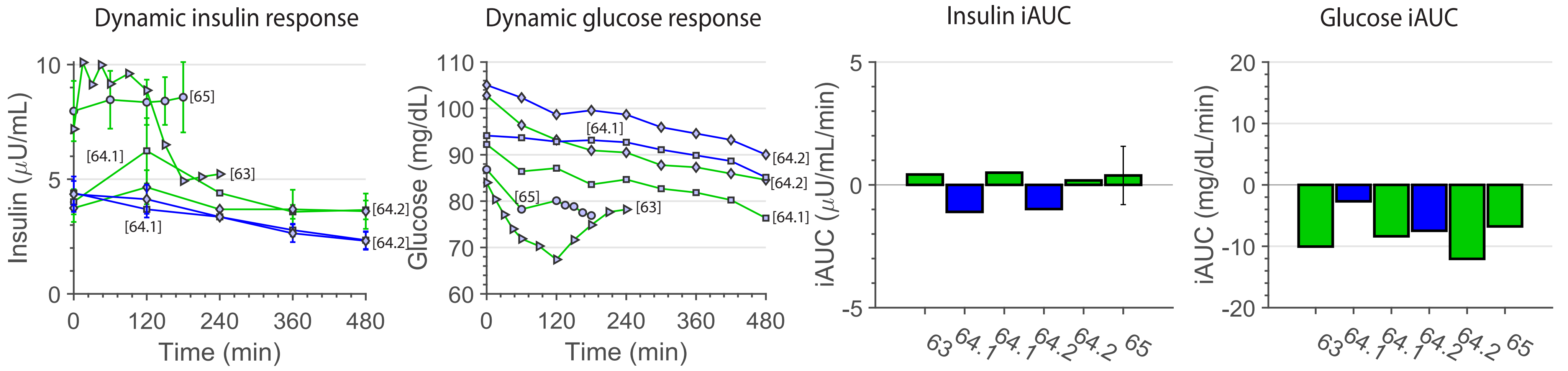

*iAUC: Incremental area under the curve
**iAUC, time to peak and peak concentrations were calculated from the extracted data
