# Supplementary material for: The Impact of Amino Acids on Postprandial Glucose and Insulin Kinetics in Humans: A Quantitative Overview
Source: Nutrients. 2020 Oct 21;12(10):3211. doi: 10.3390/nu12103211 (PMC7594055; doi:10.3390/nu12103211)
Supplement: Supplementary file 1 [file nutrients-12-03211-s001.zip › Supplemental Figure 1.pdf]

Effect of isoleucine intake on glucose and insulin dynamics

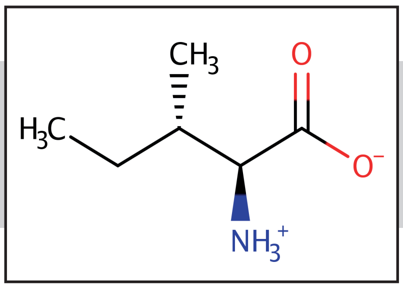

A — Oral isoleucine ingestion

First author	Subject condition	Amount administred	Ingestion type	# Subjects	Insulin time points	Insulin time to peak (min)	Insulin peak concentration (μU/mL)	Glucose time points	Glucose time to peak (min)	Glucose peak concentration (mg/dL)
[18] Nuttal, FQ	Healthy	Water	Oral	9	14	20	11.5	14	60	83.1
	Healthy	7.4g isoleucine	Oral	9	14	40	12.4	14	0	84.1

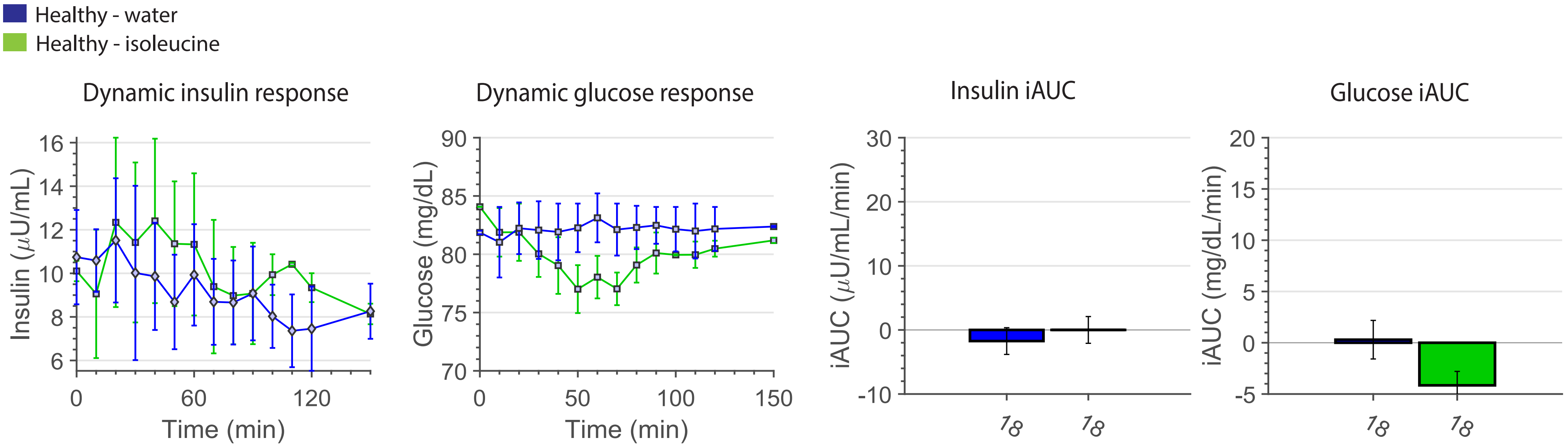

B — Oral isoleucine+glucose ingestion

First author	Subject condition	Amount administred	Ingestion type	# Subjects	Insulin time points	Insulin time to peak (min)	Insulin peak concentration (μU/mL)	Glucose time points	Glucose time to peak (min)	Glucose peak concentration (mg/dL)
[18] Nuttal, FQ	Healthy	25g glucose	Oral	9	14	40	47.2	14	40	119.9
	Healthy	7.4g isoleucine +25g glucose	Oral	9	14	40	70.9	14	30	114.1

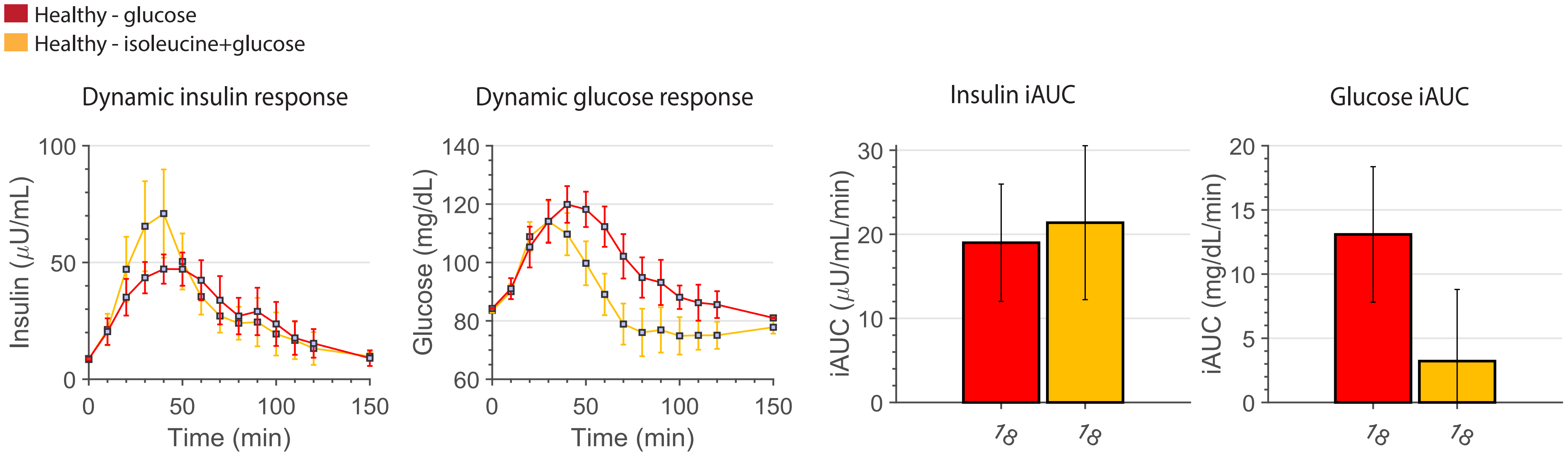

C — Intravenous isoleucine infusion

First author	Subject condition	Amount administred	Ingestion type	# Subjects	Insulin time points	Insulin time to peak (min)	Insulin peak concentration (μU/mL)	Glucose time points	Glucose time to peak (min)	Glucose peak concentration (mg/dL)
No Data	No Data	No Data	No Data	No Data	No Data	No Data	No Data	No Data	No Data	No Data

*iAUC: Incremental area under the curve

**iAUC, time to peak and peak concentrations were calculated from the extracted data
